# Navigating Hemorrhagic Shock: Biomarkers, Therapies, and Challenges in Clinical Care

**DOI:** 10.3390/biomedicines12122864

**Published:** 2024-12-17

**Authors:** Kenneth Meza Monge, Caleb Rosa, Christopher Sublette, Akshay Pratap, Elizabeth J. Kovacs, Juan-Pablo Idrovo

**Affiliations:** 1 Department of Surgery, Division of G.I, Trauma, and Endocrine Surgery, University of Colorado, Aurora, CO 80045, USA; kenneth.mezamonge@cuanschutz.edu (K.M.M.); caleb.rosa@ucdenver.edu (C.R.); christopher.sublette@cuanschutz.edu (C.S.); akshay.chauhan@cuanschutz.edu (A.P.); elizabeth.kovacs@cuanschutz.edu (E.J.K.); 2 Department of Immunology and Microbiology, University of Colorado, Aurora, CO 80045, USA

**Keywords:** hemorrhagic shock, trauma resuscitation, inflammation, coagulopathy, damage control

## Abstract

Hemorrhagic shock remains a leading cause of preventable death worldwide, with mortality patterns varying significantly based on injury mechanisms and severity. This comprehensive review examines the complex pathophysiology of hemorrhagic shock, focusing on the temporal evolution of inflammatory responses, biomarker utility, and evidence-based therapeutic interventions. The inflammatory cascade progresses through distinct phases, beginning with tissue injury and endothelial activation, followed by a systemic inflammatory response that can transition to devastating immunosuppression. Recent advances have revealed pattern-specific responses between penetrating and blunt trauma, necessitating tailored therapeutic approaches. While damage control resuscitation principles and balanced blood product administration have improved outcomes, many molecular targeted therapies remain investigational. Current evidence supports early hemorrhage control, appropriate blood product ratios, and time-sensitive interventions like tranexamic acid administration. However, challenges persist in biomarker validation, therapeutic timing, and implementation of personalized treatment strategies. Future directions include developing precision medicine approaches, real-time monitoring systems, and novel therapeutic modalities while addressing practical implementation barriers across different healthcare settings. Success in hemorrhagic shock management increasingly depends on integrating multiple interventions across different time points while maintaining focus on patient-centered outcomes.

## 1. Introduction

Hemorrhagic shock represents one of the most challenging and lethal conditions in critical care medicine, with mortality patterns that vary significantly based on both the mechanism and severity of blood loss [1]. While accounting for 30–40% of trauma-related deaths worldwide and causing approximately 60,000 deaths annually in the United States alone, the clinical presentation and underlying pathophysiology differ markedly between penetrating trauma with rapid exsanguination versus blunt trauma with tissue destruction and extensive tissue factor release [2]. Understanding these pattern-specific responses is crucial for appropriate therapeutic intervention and improved patient outcomes. The severity of hemorrhagic shock follows a well-established classification system that directly influences both the inflammatory response and therapeutic approach [3]. Class I hemorrhage (blood loss <15% of total volume) typically triggers a minimal systemic response, while Class II hemorrhage (15–30% loss) initiates compensatory mechanisms, including sympathetic activation and tachycardia. Class III hemorrhage (30–40% loss) represents a critical threshold where inflammatory cascades become prominently activated, and tissue hypoperfusion becomes clinically apparent through markers such as base deficit and lactate elevation [4,5]. Class IV hemorrhage (>40% loss) typically presents with profound shock, marked inflammatory activation, and rapid progression to organ dysfunction without immediate intervention [6].

**Figure 1:**
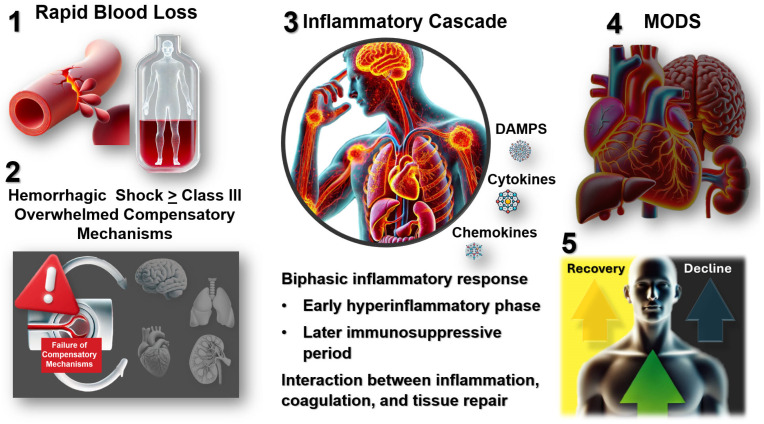
Pathophysiological Progression of Hemorrhagic Shock and Its Systemic Impact. This figure provides an overview of the sequential events underlying hemorrhagic shock, beginning with rapid blood loss, which initiates systemic hypoperfusion and compromises oxygen delivery. As blood loss exceeds compensatory thresholds (≥Class III hemorrhage), physiological mechanisms such as tachycardia and vasoconstriction fail, leading to widespread tissue hypoxia and organ dysfunction. This failure triggers a biphasic inflammatory response characterized by an initial hyperinflammatory phase, driven by damage-associated molecular patterns (DAMPs), cytokines, and chemokines, followed by a subsequent immunosuppressive phase that exacerbates tissue injury and impairs recovery. The unchecked progression of inflammation and hypoperfusion often culminates in multi-organ dysfunction syndrome (MODS), with failure of vital organs such as the brain, heart, liver, and kidneys. Clinically, outcomes diverge depending on the timing and adequacy of therapeutic interventions. Prompt and targeted treatments may promote recovery, while delayed or insufficient management results in further decline and increased mortality. Abbreviations: DAMPs (Damage-Associated Molecular Patterns), MODS (Multi-Organ Dysfunction Syndrome).

The pathophysiology of hemorrhagic shock extends far beyond simple blood volume loss, encompassing a complex cascade of inflammatory responses that evolves through distinct temporal phases [7]. The initial response focuses on hemorrhage control and damage control resuscitation, emphasizing the principles of limited crystalloid administration, appropriate blood product ratios, and permissive hypotension in selected patients [8]. This phase transitions into a systemic inflammatory response that, if inadequately managed, often proves more lethal than the original injury. Understanding this progression from compensated shock through inflammatory activation to potential immunosuppression represents a crucial challenge in improving patient outcomes. Recent advances in molecular biology and clinical research have revealed that the inflammatory response in hemorrhagic shock follows a biphasic pattern, with an initial hyperinflammatory phase followed by a potentially devastating immunosuppressive period [9]. This complex temporal evolution creates unique challenges for therapeutic intervention, as treatments beneficial during one phase may prove harmful during another. Furthermore, interactions between inflammation, coagulation, and tissue repair create a dynamic pathophysiological environment that requires carefully timed and precisely targeted interventions based on objective clinical and laboratory parameters [10] (Figure 1).

Despite significant advances in resuscitation strategies and critical care medicine, mortality rates from hemorrhagic shock remain unacceptably high, particularly in cases of severe tissue injury combined with major blood loss [11]. Traditional approaches focusing solely on volume replacement and hemodynamic stabilization fail to address the underlying inflammatory cascade that often determines patient outcomes [12]. While the emergence of molecular targeted therapies and sophisticated monitoring technologies offers new possibilities for improving survival, clinical implementation requires careful validation and standardization of care protocols based on injury patterns and shock severity. This comprehensive review examines the intricate pathophysiology of inflammation in hemorrhagic shock, focusing on molecular mechanisms, clinically validated biomarkers, and therapeutic interventions supported by current evidence. We begin by exploring the complex cascade of inflammatory mediators and cellular responses that characterize different injury patterns in hemorrhagic shock, followed by an analysis of practical biomarkers for patient monitoring and risk stratification. The review then evaluates therapeutic strategies targeting specific aspects of the inflammatory response, emphasizing approaches with demonstrated clinical efficacy while objectively assessing experimental interventions. Throughout, we emphasize the translation of basic science discoveries into practical clinical applications, maintaining a balanced perspective on the current state of evidence and future directions in the field.

## 2. Pathophysiology and Clinical Progression of Hemorrhagic Shock

**Figure 2:**
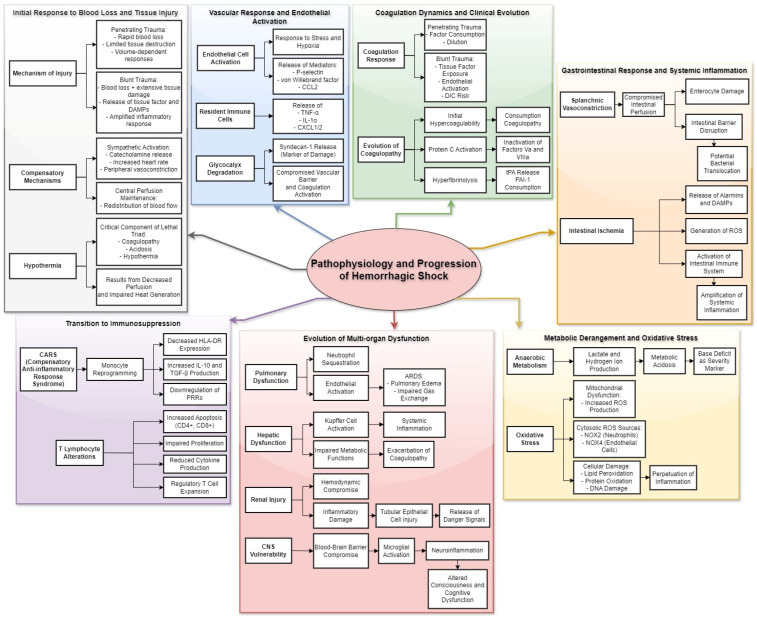
Pathophysiology and Progression of Hemorrhagic Shock. This diagram summarizes the interconnected pathophysiological mechanisms underlying hemorrhagic shock, detailing the progression from initial injury to systemic complications. The initial response to blood loss and tissue injury varies by trauma type. Penetrating trauma involves rapid blood loss with limited tissue destruction, while blunt trauma combines blood loss with significant tissue damage, amplifying inflammation. Early compensatory mechanisms, including catecholamine release, maintain central perfusion at the expense of non-vital organs. Hypothermia, a critical component of the “lethal triad,” results from decreased perfusion and impaired metabolic heat generation. The vascular response and endothelial activation involve endothelial cells responding to stress and hypoxia by releasing P-selectin and von Willebrand factor, while resident immune cells secrete TNF-α, IL-1α, and neutrophil-attracting chemokines (CXCL1/2). Glycocalyx degradation releases syndecan-1, compromising vascular integrity and promoting coagulation and inflammation. The coagulation response differs by injury pattern. Penetrating trauma leads to factor consumption and dilution, whereas blunt trauma triggers disseminated intravascular coagulation through extensive tissue factor release and endothelial activation. Hypercoagulability evolves into consumption coagulopathy, driven by Protein C activation and hyperfibrinolysis. The gastrointestinal response highlights shock-induced splanchnic vasoconstriction, which compromises intestinal perfusion, leading to enterocyte damage and barrier dysfunction. This disruption allows bacterial translocation and the release of inflammatory mediators such as DAMPs and ROS, amplifying systemic inflammation. Metabolic derangements and oxidative stress reflect the shift to anaerobic metabolism, generating lactate and hydrogen ions, contributing to metabolic acidosis. Mitochondrial dysfunction and ROS production from NADPH oxidase isoforms (NOX2/NOX4) result in cellular damage, including lipid peroxidation and DNA injury, perpetuating inflammation. The evolution of multi-organ dysfunction follows a predictable pattern. Pulmonary dysfunction arises early due to neutrophil sequestration and endothelial activation, progressing to ARDS. Hepatic dysfunction stems from hypoperfusion and Kupffer cell activation, impairing metabolic functions and exacerbating coagulopathy. Renal injury involves microvascular dysfunction and tubular epithelial damage, while CNS injury results from blood-brain barrier compromise, microglial activation, and neuroinflammation, manifesting as altered consciousness. Finally, the transition to immunosuppression is characterized by the compensatory anti-inflammatory response syndrome (CARS). Monocyte reprogramming reduces HLA-DR expression and antigen presentation, while T lymphocyte populations undergo apoptosis and impaired cytokine production. Regulatory T cell expansion further suppresses immune responses, increasing vulnerability to infection. Abbreviations: DAMPs (Damage-Associated Molecular Patterns), ROS (Reactive Oxygen Species), TNF-α (Tumor Necrosis Factor Alpha), IL-1α (Interleukin 1 Alpha), CXCL1/2 (C-X-C Motif Ligands 1 and 2), ARDS (Acute Respiratory Distress Syndrome), CARS (Compensatory Anti-inflammatory Response Syndrome), HLA-DR (Human Leukocyte Antigen DR), tPA (Tissue Plasminogen Activator), PAI-1 (Plasminogen Activator Inhibitor-1), NOX2/NOX4 (NADPH Oxidase Isoforms).

The pathophysiological response to hemorrhagic shock represents a complex interplay between injury pattern, blood loss severity, and temporal progression of the inflammatory response [13]. Understanding these interactions requires careful consideration of both the immediate consequences of tissue injury and blood loss, as well as the subsequent cascade of inflammatory events that determine patient outcomes (Figure 2).

### 2.1. Initial Response to Blood Loss and Tissue Injury

The physiological response to hemorrhagic shock varies significantly based on both the mechanism and extent of tissue injury. Penetrating trauma typically presents with rapid blood loss but relatively limited tissue destruction, triggering primarily volume-dependent responses [14]. By contrast, blunt trauma combines blood loss with extensive tissue damage, releasing substantial amounts of tissue factor and damage-associated molecular patterns (DAMPs) that amplify the inflammatory response [15]. This distinction is crucial for understanding the immediate clinical presentation and subsequent therapeutic requirements. The magnitude of blood loss dictates the initial compensatory response. Early compensatory mechanisms include sympathetic activation with subsequent catecholamine release, leading to increased heart rate and peripheral vasoconstriction [16]. These responses maintain central perfusion by redistributing blood flow and sacrificing perfusion to non-vital organs to preserve blood flow to the heart and brain. Plasma epinephrine and norepinephrine levels increase dramatically during this period, driving the characteristic tachycardia and vasoconstriction of early hemorrhagic shock [17].

Temperature regulation becomes compromised early in hemorrhagic shock, with hypothermia representing a critical component of the lethal triad alongside coagulopathy and acidosis [18]. This hypothermia results from decreased tissue perfusion and impaired metabolic heat generation rather than from inflammatory cytokine effects on thermoregulation [19]. Thus, prevention of hypothermia represents a crucial early intervention in hemorrhagic shock management, particularly in patients requiring massive transfusion.

### 2.2. Vascular Response and Endothelial Activation

The vascular endothelium is a primary sensor and critical mediator of the shock response [20]. Endothelial cells respond to mechanical stress, hypoxia, and inflammatory signals through a coordinated series of molecular events [21]. These cells rapidly mobilize preformed inflammatory mediators, including P-selectin and von Willebrand factor, while initiating synthesis of additional factors such as chemokine C-C motif ligand 2 (CCL2) [22]. Simultaneously, resident immune cells within the vessel wall, particularly tissue macrophages and mast cells, release tumor necrosis factor alpha (TNF-α), interleukin 1 alpha (IL-1α), and the neutrophil-attracting chemokines C-X-C motif ligand 1 and 2 (CXCL1/2) [23,24]. The endothelial glycocalyx, a complex carbohydrate-rich layer lining the vascular lumen, undergoes rapid degradation during hemorrhagic shock [25]. This process releases syndecan-1 into circulation, serving as a specific marker of glycocalyx damage rather than general endothelial dysfunction [26]. The loss of glycocalyx integrity compromises vascular barrier function and exposes the underlying endothelium, promoting inflammatory cell adhesion and activation of coagulation pathways through tissue factor exposure [27].

### 2.3. Gastrointestinal Response and Systemic Inflammation

The gastrointestinal tract plays a central and potentially driving role in the progression of hemorrhagic shock, representing a critical but often underappreciated mediator of systemic inflammation [28]. Early shock-induced splanchnic vasoconstriction rapidly compromises intestinal perfusion, leading to enterocyte damage and disruption of intestinal barrier function. This barrier dysfunction may permit bacterial translocation, although the precise circumstances under which this occurs and its causal relationship to outcomes remain areas of active investigation [29]. Experimental evidence suggests that enterectomy can dramatically improve survival in hemorrhagic shock models, highlighting the gut’s crucial role in driving inflammatory progression [30]. Intestinal ischemia triggers a cascade of local and systemic inflammatory responses [31,32,33].

Damaged enterocytes release alarmins and other damage-associated molecular patterns, while hypoxic intestinal epithelium generates reactive oxygen species (ROS) through both mitochondrial dysfunction and activation of cellular oxidases. The intestinal immune system, particularly resident macrophages and dendritic cells, responds to these danger signals by producing pro-inflammatory cytokines and chemokines [34]. This intestinal inflammatory response can amplify systemic inflammation through multiple pathways, including direct release of inflammatory mediators into the portal circulation and activation of gut-associated lymphoid tissue.

### 2.4. Metabolic Derangement and Oxidative Stress

Cellular metabolic dysfunction in hemorrhagic shock reflects decreased oxygen delivery and intrinsic alterations in cellular metabolism [7]. The shift from aerobic to anaerobic metabolism generates lactate and hydrogen ions, contributing to metabolic acidosis. This acidosis, quantified through base deficit measurement, serves as a marker of shock severity and a guide for resuscitation adequacy [35]. Notably, both lactate levels and base deficit are routinely monitored in clinical practice, providing real-time feedback on tissue perfusion and resuscitation effectiveness. Oxidative stress emerges as a key mediator of cellular damage in hemorrhagic shock. Mitochondrial dysfunction leads to increased production of ROS, particularly from complexes I and III of the electron transport chain [36]. Cytosolic sources of oxidative stress include NADPH oxidase 2 (NOX2) in neutrophils and NOX4 in endothelial cells. This oxidative stress triggers widespread cellular damage through lipid peroxidation, protein oxidation, and DNA damage, particularly affecting mitochondrial DNA [37,38,39]. The resulting cellular damage perpetuates the inflammatory response by releasing oxidized cellular components that serve as additional danger signals.

### 2.5. Evolution of Multi-Organ Dysfunction

The progression from localized tissue injury to multi-organ dysfunction follows a predictable yet complex temporal sequence [40]. The lungs typically manifest early dysfunction due to their extensive capillary network and resident immune cell population. Neutrophil sequestration within the pulmonary vasculature, combined with endothelial activation, can progress to acute respiratory distress syndrome characterized by protein-rich pulmonary edema and impaired gas exchange [41,42]. Hepatic dysfunction develops through both direct hypoxic injury and inflammatory mediator effects [43]. Kupffer cells, the liver’s resident macrophages, become activated and contribute to systemic inflammation through cytokine production. The combination of reduced hepatic perfusion and inflammatory mediators impairs normal metabolic functions, including glucose regulation and drug metabolism [44,45]. This hepatic dysfunction can exacerbate coagulopathy through decreased production of both pro- and anti-coagulant factors.

Renal injury manifests through a combination of hemodynamic compromise and inflammatory damage [46]. Glomerular filtration becomes impaired not only through systemic hypotension but also through local microvascular dysfunction mediated by endothelial activation and microthrombus formation [47]. Tubular epithelial cells, damaged by ischemia and inflammatory mediators, release additional danger signals that perpetuate local inflammation while contributing to systemic inflammatory mediator levels [48]. The central nervous system is particularly vulnerable to the combined effects of hypoperfusion and inflammation [49]. The blood–brain barrier becomes compromised as circulating TNF-α and IL-1β activate brain endothelial cells and weaken tight junctions [50]. This barrier dysfunction permits the infiltration of inflammatory cells and mediators into the brain parenchyma, triggering microglial activation and neuroinflammation [51]. These changes manifest clinically as altered consciousness and cognitive dysfunction, potentially contributing to long-term neurological sequelae in survivors.

### 2.6. Coagulation Dynamics and Clinical Evolution

The coagulation response in hemorrhagic shock reflects complex interactions between tissue injury pattern, shock severity, and inflammatory activation [52]. In penetrating trauma, the primary driver of coagulopathy typically involves factor consumption and dilution [53]. However, blunt trauma introduces extensive tissue factor exposure and widespread endothelial activation, potentially triggering disseminated intravascular coagulation [54]. This pattern-specific understanding proves crucial for appropriate hemostatic resuscitation strategies.

Thromboelastography (TEG) and rotational thromboelastometry (ROTEM) provide valuable information about real-time coagulation status, although their utility faces certain limitations [55]. While these technologies offer significant advantages over traditional coagulation testing, their measurements reflect past conditions and may not capture rapidly evolving changes in coagulation status. Additionally, these assays cannot directly assess endothelial contributions to coagulation dysfunction [56]. Nevertheless, they remain valuable tools for guiding blood product administration and identifying specific coagulation abnormalities requiring targeted intervention. The evolution of trauma-induced coagulopathy often follows a predictable sequence but can vary markedly based on injury pattern and shock severity. Initial hypercoagulability, driven by tissue factor release and platelet activation, may progress to consumption coagulopathy as clotting factors become depleted [57]. Protein C activation, triggered by the thrombin-thrombomodulin complex on endothelial cells, can further complicate this picture by inactivating factors Va and VIIIa [58,59]. Simultaneously, hyperfibrinolysis may develop as tissue plasminogen activator releases from damaged endothelium while its primary inhibitor, plasminogen activator inhibitor-1, becomes consumed or degraded [60].

### 2.7. Transition to Immunosuppression

The progression from initial hyperinflammation to subsequent immunosuppression represents a critical transition point in hemorrhagic shock pathophysiology [61]. This compensatory anti-inflammatory response syndrome (CARS) reflects complex reprogramming of immune system function rather than simple exhaustion [62]. Understanding this transition proves crucial for anticipating and potentially preventing late complications, particularly infectious complications. The immunosuppressive phase manifests through specific cellular and molecular changes. Monocytes undergo reprogramming characterized by decreased human leukocyte antigen DR (HLA-DR) expression and reduced antigen presentation capacity [63,64]. This change occurs through epigenetic modifications affecting promoter regions of pro-inflammatory genes. Simultaneously, these cells increase the production of anti-inflammatory mediators, notably interleukin 10 (IL-10) and transforming growth factor beta (TGF-β), while reducing their responsiveness to inflammatory stimuli through the downregulation of pattern recognition receptors [65,66].

T lymphocyte populations undergo substantial alterations during this phase. The conventional T cell population experiences increased apoptosis, particularly affecting CD4+ and CD8+ effector populations, leading to lymphopenia [67,68]. The remaining T cells show impaired proliferation in response to antigens and decreased production of pro-inflammatory cytokines, especially interferon gamma and interleukin 2 [69]. Simultaneously, the regulatory T cell population expands, actively suppressing immune responses through contact-dependent mechanisms and secretion of immunosuppressive cytokines [70].

### 2.8. Clinical Implications and Therapeutic Windows

The temporal evolution of hemorrhagic shock creates distinct therapeutic windows requiring specific intervention strategies [3]. The initial phase demands focus on hemorrhage control and appropriate resuscitation, with careful attention to preventing hypothermia, acidosis, and coagulopathy. This period typically spans the first few hours after injury, during which damage control principles prove crucial for optimal outcomes [71]. The subsequent inflammatory phase presents opportunities for targeted intervention but requires careful balance. While excessive inflammation can drive organ dysfunction, some degree of inflammatory response remains necessary for proper wound healing and antimicrobial defense [72]. Biomarker guidance during this phase may help to identify patients at particular risk for inflammatory complications, though the clinical utility of many proposed markers remains under investigation. The transition to immunosuppression creates a particularly vulnerable period requiring careful monitoring for infectious complications. This phase typically emerges 3–5 days after the initial injury and may persist for weeks in severely injured patients [73]. Recognition of this immunosuppressive state by monitoring monocyte HLA-DR expression or other markers may help to guide antimicrobial prophylaxis and immunomodulatory interventions [74]. However, the optimal approach to managing this phase remains an area of active investigation.

## 3. Biomarkers and Predictors of Inflammatory Outcomes in Hemorrhagic Shock

The complex pathophysiology of hemorrhagic shock necessitates reliable biomarkers for monitoring disease progression and guiding therapeutic interventions. While numerous potential biomarkers have been identified through research, their clinical utility requires careful consideration of availability, timeliness, and validated correlation with patient outcomes [75]. (Table 1).

### 3.1. Established Clinical Markers of Shock Severity

Base deficit and lactate measurements represent cornerstone assessments in hemorrhagic shock, providing essential information about tissue perfusion and metabolic derangement [76]. These markers are routinely monitored in clinical practice, offering real-time feedback about shock severity and resuscitation adequacy. Base deficit values exceeding 6 mmol/L indicate severe shock and correlate strongly with mortality risk, while lactate elevation above 4 mmol/L signals significant tissue hypoperfusion [77]. The trajectory of these markers during resuscitation often proves more valuable than absolute values, with failure to normalize within 24 h indicating inadequate resuscitation or ongoing tissue injury. Viscoelastic testing through thromboelastography (TEG) or rotational thromboelastometry (ROTEM) provides crucial information about coagulation status [55]. These technologies offer advantages over traditional coagulation testing in assessing clot formation dynamics and strength, providing real-time feedback for therapeutic adjustments. Key parameters include clot formation time, maximum amplitude, and fibrinolysis, each offering specific insights into coagulation abnormalities [78]. Technology enables rapid identification of specific hemostatic deficiencies, guiding targeted blood product administration. However, their utility faces certain constraints, including the inherent delay between sampling and results, the potential disparity between in vitro findings and in vivo coagulation status, and the inability to directly assess endothelial contributions to coagulopathy [56]. Understanding these limitations while recognizing the value of viscoelastic testing remains crucial for optimal utilization in hemorrhagic shock management.

### 3.2. Endothelial Dysfunction Assessment

Endothelial dysfunction biomarkers provide critical insight into vascular barrier integrity and endothelial activation status. Syndecan-1 specifically indicates glycocalyx degradation, with elevated plasma levels correlating with mortality risk and massive transfusion requirements [79]. The marker’s utility extends beyond simple prognostication, offering insights into the timing and severity of endothelial glycocalyx disruption. This information proves particularly valuable in understanding the progression from compensated to decompensated shock states [27,80]. Von Willebrand factor activity, modulated by ADAMTS13, reflects another crucial aspect of endothelial response to injury [75]. Decreased ADAMTS13 activity in severe shock indicates compromised vascular homeostasis, while elevated von Willebrand factor levels signal endothelial activation [81,82]. The balance between these factors influences both coagulation status and inflammatory progression. While these markers currently serve primarily research purposes due to availability and processing time constraints, their mechanistic insights inform therapeutic strategies targeting endothelial protection.

### 3.3. Inflammatory Mediator Profiles

The role of inflammatory cytokines in hemorrhagic shock presents a complex picture requiring careful interpretation. While numerous studies demonstrate elevation of classic inflammatory mediators such as TNF-α and interleukin 6 (IL-6), their concentrations typically remain significantly lower than in septic patients [83,84]. This observation helps to explain the limited success of anti-cytokine therapies in hemorrhagic shock despite their theoretical appeal. IL-6 measurements during the first 24–72 h post-injury demonstrate correlations with trauma severity and mortality risk, although their clinical utility faces practical limitations [85]. The marker’s predictive value for multiple organ dysfunction development exists primarily in research settings, as the time required for analysis often exceeds the window for therapeutic intervention.

Additional cytokines, including IL-8 and IL-10, may predict complications but rarely alter acute clinical management [86]. These markers help to characterize the transition from pro-inflammatory to anti-inflammatory phases, although their practical utility remains constrained by measurement limitations and timing considerations.

### 3.4. Traditional Acute Phase Proteins

While widely available, C-reactive protein (CRP) and procalcitonin demonstrate limited utility in acute hemorrhagic shock management [87,88]. Both markers show considerable overlap in their elevation patterns and often provide little information beyond what is clinically apparent. Procalcitonin, in particular, frequently shows marked elevation (>20 ng/mL) in critically ill patients, making it less useful for distinguishing infectious complications from sterile inflammation [89,90]. These markers retain value for monitoring recovery and identifying late complications, particularly in the setting of suspected infections or inflammatory complications.

### 3.5. Emerging Molecular Markers

Recent advances in molecular biology have identified numerous potential biomarkers for hemorrhagic shock, although their translation to clinical practice requires careful validation. Cell-free DNA, particularly mitochondrial DNA, represents one such candidate [91]. Released during cellular damage, these molecules serve as danger signals that can amplify inflammatory responses. While cell-free DNA levels correlate with injury severity and predict inflammatory complications in research settings, technical challenges in measurement and standardization currently limit their clinical application [92].

High mobility group box 1 (HMGB1) has emerged as an important damage-associated molecular pattern linking cellular damage to inflammatory response [93]. Released from damaged cells, HMGB1 levels correlate with injury severity and organ dysfunction development in experimental models [94]. However, like many molecular markers, HMGB1 measurement faces practical constraints, including processing time and standardization challenges.

MicroRNA profiles represent another frontier in biomarker development, though their current utility remains primarily investigational [95] Specific microRNAs, such as miR-146a and miR-155, regulate Toll-like receptor signaling and inflammatory responses [96]. While altered expression patterns of these molecules correlate with inflammatory response magnitude and subsequent organ dysfunction, technical complexity and cost of measurement currently restrict their use to research applications.

### 3.6. Integration of Multiple Markers for Clinical Decision-Making

The complexity of hemorrhagic shock pathophysiology suggests that no single biomarker can comprehensively assess patient status or prognosis [97]. Current evidence supports an integrated approach combining established clinical markers with selective use of newer molecular indicators. Base deficit and lactate measurements, combined with viscoelastic testing results, provide the foundation for acute clinical decision making [98,99]. Additional markers may offer supplementary information in specific clinical scenarios, though their utility must be weighed against practical considerations of cost, processing time, and availability.

The timing of biomarker measurement proves crucial for meaningful interpretation and clinical application. Early markers provide immediate feedback about resuscitation adequacy, while later markers help identify developing complications [100,101]. Understanding these temporal relationships enables clinicians to select appropriate markers for specific clinical questions while avoiding unnecessary testing.

## 4. Therapeutic Interventions in Hemorrhagic Shock

**Figure 3:**
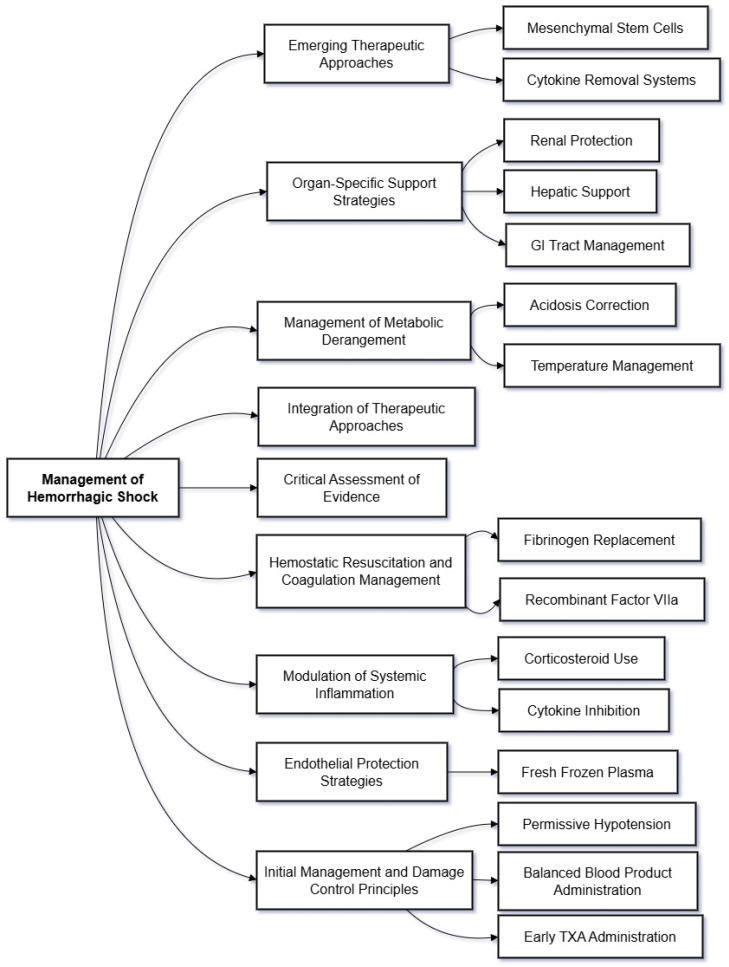
Therapeutic Interventions in Hemorrhagic Shock. This diagram outlines key therapeutic strategies employed in managing hemorrhagic shock, organized by intervention type. It highlights established treatments and emerging approaches, emphasizing the importance of timing, integration, and evidence-based practices in optimizing outcomes. The Initial Management and Damage Control Principles prioritize permissive hypotension, balanced blood product administration in a 1:1:1 ratio, and early tranexamic acid (TXA) administration to control bleeding, mitigate coagulopathy, and stabilize patients during the acute phase. Endothelial Protection Strategies involve administering fresh frozen plasma to stabilize vascular barriers and reduce glycocalyx degradation, although targeted therapies remain largely experimental. Modulation of Systemic Inflammation explores the limited role of corticosteroids and cytokine inhibition, given the complexity and redundancy of inflammatory signaling pathways. Hemostatic Resuscitation and Coagulation Management focuses on fibrinogen replacement and recombinant activated factor VIIa (rFVIIa) for severe coagulopathy, while emphasizing the need for early intervention to prevent coagulopathy progression. The Management of Metabolic Derangement underscores the importance of acidosis correction through effective resuscitation and hemorrhage control, alongside early temperature management to prevent the deleterious cycle of hypothermia and coagulopathy. Organ-specific support Strategies address renal protection, hepatic support, and gastrointestinal tract management, focusing on maintaining perfusion and minimizing further injury to vital organs. Emerging Therapeutic Approaches, including mesenchymal stem cell therapy and extracorporeal cytokine removal systems, remain investigational but have the potential to modulate immune responses and mitigate systemic inflammation. Finally, the Integration of Therapeutic Approaches highlights the need for coordinated, phase-specific interventions that adapt to patient-specific injury patterns and shock severity. This comprehensive management framework balances competing priorities, such as fluid restriction for pulmonary protection versus maintaining renal perfusion, while incorporating standard physiologic and biomarker assessments to guide therapeutic decisions. Abbreviations: TXA (Tranexamic Acid), rFVIIa (Recombinant Activated Factor VIIa), GI (Gastrointestinal).

The management of hemorrhagic shock requires a coordinated approach that prioritizes proven interventions while carefully evaluating emerging therapeutic options [3]. Current evidence strongly supports early hemorrhage control and appropriate resuscitation as foundational interventions, while the utility of targeted molecular therapies remains largely investigational [102]. (Figure 3) (Table 2).

### 4.1. Initial Management and Damage Control Principles

The immediate management of hemorrhagic shock focuses on hemorrhage control and appropriate resuscitation, following well-established damage control principles [140]. Permissive hypotension, maintaining systolic blood pressure between 80 and 90 mmHg without traumatic brain injury, helps to prevent dilutional coagulopathy and reduces the inflammatory response triggered by excessive crystalloid administration [103,104]. This strategy requires careful patient selection and monitoring, particularly injury patterns and comorbidities.

Blood product administration follows specific ratios based on substantial clinical evidence, particularly from the Pragmatic, Randomized Optimal Platelet and Plasma Ratios (PROPPR) trial [105]. The standard 1:1:1 ratio of packed red blood cells, fresh frozen plasma, and platelets provides superior outcomes compared to historical approaches [106]. This balanced resuscitation strategy addresses volume replacement and coagulation factor deficiency while potentially modulating the inflammatory response through plasma-mediated effects on endothelial function.

Early tranexamic acid (TXA) administration represents another evidence-based intervention supported by the Clinical Randomization of an Antifibrinolytic in Significant Hemorrhage (CRASH-2) trial [107]. TXA administration within three hours of injury reduces mortality through multiple mechanisms, including direct inhibition of plasminogen activation, preservation of platelet function, and modulation of inflammatory responses through reduced complement activation and decreased neutrophil activation [108,109,141]. The timing-dependent effects reflect both the evolution of fibrinolytic processes and the changing inflammatory milieu, with delayed administration potentially increasing mortality through paradoxical enhancement of thrombotic risk.

### 4.2. Endothelial Protection Strategies

Fresh frozen plasma administration provides endothelial protection beyond its hemostatic effects, though the mechanisms remain incompletely understood [110]. Plasma appears to reduce glycocalyx shedding and stabilize endothelial barrier function through multiple mechanisms, including direct protein supplementation and modulation of inflammatory mediators [111,112]. While clinical evidence supports early plasma administration in high ratios relative to red blood cells, the specific endothelial protective effects in human patients require further investigation.

Targeted endothelial protection strategies beyond plasma administration remain largely experimental. While numerous compounds have shown promise in preclinical studies, translation to clinical practice has proved challenging due to the complex nature of endothelial dysfunction and difficulties in delivering therapeutic agents to damaged endothelium.

### 4.3. Modulation of Systemic Inflammation

The role of corticosteroids in hemorrhagic shock remains controversial, with current evidence suggesting limited utility in the acute phase [113,114]. While low-dose hydrocortisone administration has theoretical benefits for modulating inflammatory responses, clinical trials have failed to demonstrate consistent benefits. The administration of corticosteroids may prove more appropriate for managing post-shock septic complications rather than as primary therapy for hemorrhagic shock itself.

Targeted cytokine inhibition has shown limited success in hemorrhagic shock, likely due to lower cytokine concentrations compared to septic states and the complex, redundant nature of inflammatory signaling [115,116]. Furthermore, the timing of such interventions proves crucial, as complete suppression of inflammatory responses may impair wound healing and antimicrobial defense [117].

### 4.4. Hemostatic Resuscitation and Coagulation Management

Coagulation management in hemorrhagic shock requires a careful balance between preventing excessive bleeding and avoiding thrombotic complications. Fibrinogen replacement through either cryoprecipitate or concentrate plays a crucial role in maintaining hemostasis, particularly in cases of severe tissue injury with extensive coagulation factor consumption [118]. While both sources prove effective, some evidence suggests that early, high-dose fibrinogen supplementation may improve outcomes, though definitive clinical trials are still needed [119]. The potential for fibrinogen to modulate inflammatory responses through effects on endothelial function and platelet interactions requires further investigation.

Recombinant activated factor VII (rFVIIa) remains controversial in hemorrhagic shock management [121]. While potentially beneficial in specific scenarios of refractory bleeding, its broad application has not demonstrated consistent benefit. Current evidence supports reserving rFVIIa for severe, refractory hemorrhage cases where standard hemostatic measures have proved inadequate [122].

### 4.5. Management of Metabolic Derangement

Metabolic acidosis correction requires careful attention to both cause and timing. While historically aggressive alkaline therapy was common, current evidence suggests that excessive alkali administration may prove detrimental [123]. Instead, the focus should remain on adequate tissue perfusion through appropriate resuscitation and hemorrhage control. Temperature management is crucial, with active warming measures beginning early to prevent the vicious cycle of hypothermia and coagulopathy [124,125].

### 4.6. Organ-Specific Support Strategies

Renal protection focuses primarily on maintaining adequate perfusion while avoiding nephrotoxic insults [126,137]. While numerous compounds have shown promise for preventing acute kidney injury in experimental models, none have demonstrated consistent benefit in clinical trials. Current management emphasizes appropriate resuscitation, careful medication selection, and prompt recognition of early kidney dysfunction.

Hepatic care remains largely supportive, focusing on maintaining adequate perfusion and preventing additional injury [127,128]. The liver’s crucial role in coagulation factor production and inflammatory mediator metabolism makes hepatic dysfunction particularly problematic in hemorrhagic shock [142].

The gastrointestinal tract requires particular attention given its potential role in driving inflammatory progression. Management principles include early enteral nutrition when feasible, appropriate antimicrobial prophylaxis, and careful attention to gastric pH management [129,130,131,132]. Maintaining gut barrier function may help prevent late complications, though optimal approaches require further investigation.

### 4.7. Emerging Therapeutic Approaches

Mesenchymal stem cell therapy represents a promising but still experimental approach to hemorrhagic shock management [133]. These cells can modulate immune responses and promote tissue repair through multiple mechanisms. Early clinical trials have demonstrated safety in allogeneic administration, with some evidence of improved inflammatory marker profiles [134].

Extracorporeal cytokine removal systems can effectively remove inflammatory mediators from circulation, but their impact on patient outcomes remains unclear [135]. The complexity and cost of implementation, combined with the uncertain timing of application, limit their current utility. Additional research must define appropriate patient selection criteria and optimal timing of intervention.

## 5. Challenges and Future Directions

**Figure 4:**
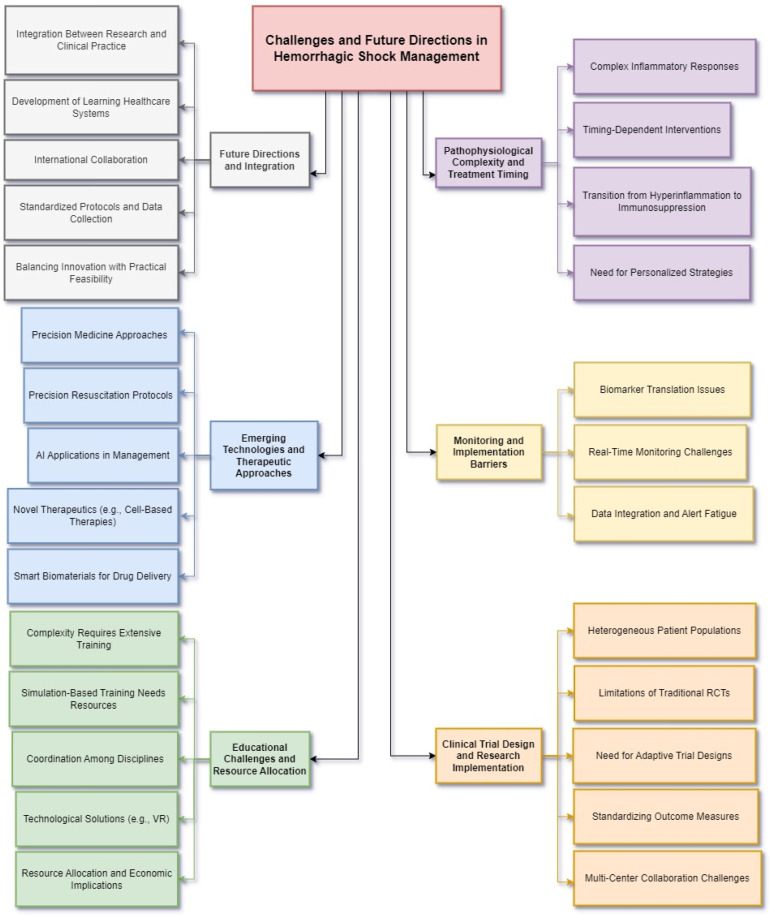
Challenges and Future Directions in Hemorrhagic Shock Management. This diagram highlights the critical challenges and emerging strategies in advancing the management of hemorrhagic shock. It emphasizes the need to address complex pathophysiological processes, improve monitoring tools, enhance clinical trial design, and integrate innovative technologies into clinical practice while overcoming resource and educational barriers. The Pathophysiological Complexity and Treatment Timing category outlines the intricate inflammatory responses and timing-dependent interventions in hemorrhagic shock. It underscores the challenges of transitioning from hyperinflammation to immunosuppression and the necessity of personalized therapeutic strategies tailored to individual patient profiles. Monitoring and Implementation Barriers focuses on issues related to translating biomarker research into practical tools for clinical use. Challenges include real-time monitoring system limitations, data integration complexities, and the risk of alert fatigue, which hinder effective implementation in acute care settings. The Clinical Trial Design and Research Implementation category addresses the difficulty of managing heterogeneous patient populations and the limitations of traditional randomized controlled trials (RCTs). Adaptive trial designs, standardized outcome measures, and multi-center collaboration are proposed as solutions to improve research efficacy and applicability. Educational Challenges and Resource Allocation emphasizes the growing complexity of hemorrhagic shock management and the need for advanced training programs, including simulation-based education and multidisciplinary coordination. Resource allocation challenges, particularly in low-resource settings, highlight the need for cost-effective yet high-quality care protocols. Emerging Technologies and Therapeutic Approaches explores the potential of precision medicine, artificial intelligence (AI) applications, novel therapeutics like cell-based therapies, and smart biomaterials for drug delivery. These innovations promise to improve patient outcomes but face barriers in implementation, including scalability, cost, and integration into acute care workflows. Finally, Future Directions and Integration advocates for closer collaboration between research and clinical practice. Priorities include the development of learning healthcare systems, international collaboration, and standardized protocols to balance innovation with practical feasibility, ensuring meaningful advancements in hemorrhagic shock management. Abbreviations: RCTs (Randomized Controlled Trials), AI (Artificial Intelligence), VR (Virtual Reality).

The translation of basic science advances into improved patient outcomes faces several critical challenges requiring coordinated solutions across research and clinical domains (Figure 4).

### 5.1. Scientific and Clinical Challenges

The complex nature of inflammatory responses in hemorrhagic shock creates fundamental challenges for therapeutic intervention [136]. Multiple parallel pathways, feedback loops, and redundant mechanisms establish a network resistant to single-target therapeutic approaches. The timing-dependent nature of various interventions, where treatments beneficial during one phase may prove harmful during another, requires sophisticated monitoring and careful therapeutic timing [3,143].

Individual variations in genetic background, pre-existing conditions, and physiological reserves create unique inflammatory profiles that may require personalized intervention strategies [144]. Response patterns vary significantly based on injury mechanism and severity, requiring careful consideration in therapeutic development. Understanding these pattern-specific responses proves crucial for appropriate therapeutic targeting [145].

### 5.2. Implementation and Monitoring Challenges

Translation of biomarker research into practical clinical tools remains problematic despite numerous promising candidates [146]. Current biomarker assessments often require sophisticated laboratory techniques with significant processing time, limiting their utility in acute decision-making [75]. The development of real-time monitoring systems capable of simultaneously tracking multiple biomarkers represents a significant technological challenge.

The integration of continuous biomarker assessment with physiological monitoring poses particular challenges in acute care settings [147]. These systems must demonstrate reliability across different clinical scenarios while providing actionable data in real time. The complexity of data interpretation in acute scenarios necessitates clear protocols for translating monitoring data into clinical decisions [148].

### 5.3. Research and Educational Challenges

Clinical trial design in hemorrhagic shock presents unique challenges requiring careful consideration of both scientific rigor and practical feasibility [138]. The heterogeneous nature of trauma patients, combined with the dynamic evolution of shock states, complicates the development of appropriate inclusion criteria and outcome measures [139].

Educational challenges include comprehensive training programs that address technical skills and clinical decision making [149]. Coordinated training across multiple specialties proves essential for optimal shock management. Resource allocation challenges affect the implementation of advanced care protocols across different healthcare settings, particularly in resource-limited environments [150].

### 5.4. Future Directions

The evolution of precision medicine approaches in hemorrhagic shock management offers promising opportunities while presenting implementation challenges. Integrating genomic and proteomic data with clinical parameters might enable more personalized therapeutic strategies, though translation to acute care settings requires careful attention to practical constraints [151].

Artificial intelligence applications show promise but require rigorous validation before clinical implementation [152]. These systems must demonstrate reliability in real-time decision support while maintaining interpretability for clinical staff. Novel therapeutic approaches focusing on tissue protection and regeneration show particular promise for improving long-term outcomes [133].

## 6. Conclusions

Managing hemorrhagic shock remains a significant challenge in modern medicine, requiring careful integration of basic science understanding with practical clinical implementation. Current evidence strongly supports early hemorrhage control and appropriate resuscitation as foundational interventions, while many molecular-targeted therapies remain investigational. Success increasingly depends on coordinating multiple interventions across different time points, carefully focusing on injury patterns and shock severity.

Future advancement requires continued research across multiple domains while focusing on practical clinical applications. Developing more precise monitoring systems and targeted therapeutic approaches shows promise for improving outcomes, though implementation challenges remain significant. International collaboration and standardization of research approaches could accelerate progress while ensuring broader applicability of findings. Success ultimately requires balancing innovation and practical feasibility while focusing on meaningful improvement in patient outcomes.

## Figures and Tables

**Table 1:** Biomarkers Assessment in Hemorrhagic Shock.

Biomarker	Type/Category	Clinical Utility	Advantages	Limitations	Current Status	References
Base Deficit	Established Clinical Marker of Shock Severity	- Indicates tissue perfusion and metabolic derangement. - Values exceeding 6 mmol/L indicate severe shock. - Correlates strongly with mortality risk.	- Routinely monitored. - Real-time feedback. - Trajectory during resuscitation is valuable.	- May not reflect localized tissue hypoperfusion. - Influenced by metabolic conditions.	Established clinical practice	[76]
Lactate	Established Clinical Marker of Shock Severity	- Signals significant tissue hypoperfusion. - Levels exceeding 4 mmol/L indicate significant hypoperfusion.	- Routinely monitored. - Real-time feedback. - Trajectory during resuscitation is important.	- Elevated in various conditions. - May be affected by liver dysfunction.	Established clinical practice	[77]
Viscoelastic Testing (TEG/ROTEM)	Established Marker of Coagulation Status	- Provides information on clot formation dynamics and strength. - Guides blood product administration.	- Advantages over traditional coagulation tests.	- Delay between sampling and results. - May not reflect in vivo status. - Does not assess endothelial contributions.	Established clinical practice	[55,78]
Syndecan-1	Marker of Glycocalyx Degradation	- Elevated levels correlate with mortality risk and massive transfusion requirements. - Indicates glycocalyx degradation.	- Provides insight into endothelial glycocalyx status.	- Primarily useful in research settings. - Limited value in routine clinical decision-making.	Research application	[27,79,80]
Von Willebrand Factor Activity/ADAMTS 13	Endothelial Dysfunction Assessment	- Reflects endothelial response to injury. - Decreased ADAMTS13 activity indicates compromised vascular homeostasis.	- Provides insight into endothelial activation.	- Limited availability and high cost. - Processing time is lengthy. - Primarily for research use.	Research application	[75,81,82]
TNF-α, IL-6	Inflammatory Mediator Profiles	- Elevated levels correlate with trauma severity and mortality risk. - IL-6 may predict multiple organ dysfunction.	- Insight into inflammatory response magnitude.	- Analysis time exceeds the therapeutic window. - Levels lower than in sepsis. - Limited acute clinical utility.	Research application	[83,84,85]
IL-8, IL-10	Inflammatory Mediator Profiles	- Levels may predict complications and inflammatory responses.	- Potential predictive value for complications.	- Rarely alter acute clinical management. - Practical limitations in measurement and timing.	Research application	[86]
C-Reactive Protein (CRP)	Traditional Acute Phase Protein	- Limited utility in acute management of hemorrhagic shock. - May monitor recovery and identify late complications.	- Widely available.	- Overlap in elevation patterns with other markers. - Provides little additional acute information.	Limited clinical utility	[87,88]
Procalcitonin	Traditional Acute Phase Protein	- May help to identify late complications. - Limited utility in acute hemorrhagic shock management.	- Widely available.	- Elevated in critically ill patients regardless of cause. - Less useful for distinguishing infections from sterile inflammation.	Limited clinical utility	[89,90]
Cell-Free DNA (cfDNA, mtDNA)	Emerging Molecular Marker	- Correlates with injury severity. - Predicts inflammatory complications. - Indicates cellular damage and acts as danger signals.	- Potential to amplify understanding of inflammatory responses.	- Technical challenges in measurement. - Standardization issues limit clinical application.	Emerging research marker	[91,92]
High Mobility Group Box 1 (HMGB1)	Emerging Molecular Marker	- Levels correlate with injury severity and organ dysfunction. - Links cellular damage to inflammatory response.	- Insight into damage-associated molecular patterns.	- Measurement constraints. - Standardization challenges. - Requires further validation for clinical use.	Emerging research marker	[93,94]
MicroRNA Profiles (e.g., miR-146a, miR-155)	Emerging Molecular Marker	- Altered expression correlates with magnitude of inflammatory response and organ dysfunction. - Regulates Toll-like receptor signaling.	- Potential for personalized therapy.	- Technical complexity and high cost. - Currently limited to research applications.	Research application	[95,96]

**Table 2:** Evidence Summary Table: Therapeutic Interventions in Hemorrhagic Shock.

Intervention	Evidence Level	Key Findings	References
Permissive Hypotension	Strong clinical evidence from multiple trials	Maintaining systolic BP between 80 and 90 mmHg without TBI helps prevent dilutional coagulopathy and reduces inflammation caused by excessive crystalloid use. Requires careful patient selection and monitoring.	[103,104]
Balanced Blood Product Administration (1:1:1 Ratio)	Strong clinical evidence (PROPPR trial)	Early administration of packed RBCs, FFP, and platelets in a 1:1:1 ratio improves outcomes by addressing volume loss and coagulation deficits and may modulate inflammation through plasma effects on endothelial function.	[105,106]
Early Tranexamic Acid (TXA) Administration	Strong evidence from CRASH-2 trial	TXA given within 3 h of injury reduces mortality via hemostatic and anti-inflammatory effects. Delayed administration (>3 h) may increase mortality, highlighting the importance of timely intervention.	[107,108,109]
Fresh Frozen Plasma for Endothelial Protection	Clinical evidence supports use; mechanisms under study	FFP not only aids hemostasis but also protects the endothelium by reducing glycocalyx shedding and stabilizing barrier function. Effects are partly due to fibrinogen and other plasma proteins. Specific endothelial protective effects in humans need further research.	[110,111,112]
Corticosteroid Use	Controversial; limited utility in acute phase	Low-dose hydrocortisone has theoretical anti-inflammatory benefits, but clinical trials show inconsistent results. May be more appropriate for managing post-shock septic complications rather than acute hemorrhagic shock.	[113,114]
Targeted Cytokine Inhibition	Limited success; experimental	Due to lower cytokine levels compared to sepsis and complex inflammatory signaling, targeted cytokine inhibition has not been effective. Timing is crucial; excessive suppression may impair wound healing and antimicrobial defense.	[115,116,117]
Fibrinogen Replacement (Cryoprecipitate/Concentrate)	Some evidence; optimal strategy under investigation	Essential for hemostasis, especially in severe injuries with coagulation factor consumption. Early, high-dose supplementation may improve outcomes, but definitive trials are needed. Potential anti-inflammatory effects via endothelial and platelet interactions require further study.	[118,119,120]
Recombinant Activated Factor VII (rFVIIa)	Controversial; limited recommendation	May benefit specific cases of refractory bleeding but lacks consistent evidence for broad use. Risks include thrombotic complications and high cost. Recommended only for severe cases unresponsive to standard measures.	[121,122]
Metabolic Acidosis Correction	Current evidence advises against aggressive alkali therapy	Excessive alkali can be harmful. Focus should be on restoring tissue perfusion through appropriate resuscitation and hemorrhage control. Base deficit improves with effective treatment and serves as a marker rather than a direct target.	[123]
Temperature Management (Preventing Hypothermia)	Strong clinical recommendation	Early active warming is crucial as preventing hypothermia is easier than correcting it. Hypothermia exacerbates coagulopathy, creating a vicious cycle that complicates resuscitation. Systematic warming protocols are essential, including warming blood products and controlling the environment.	[124,125]
Renal Protection Strategies	Emphasis on supportive care	No pharmacological interventions have shown consistent clinical benefit. Management focuses on adequate perfusion, avoiding nephrotoxins, and early recognition of kidney dysfunction.	[17,126]
Hepatic Support	Supportive care; experimental therapies unproven	Maintaining liver perfusion and preventing additional injury are key. The liver’s role in coagulation and inflammation makes dysfunction problematic. Liver support systems exist but lack evidence in acute hemorrhagic shock. Focus remains on resuscitation and careful medication use.	[127,128]
Gastrointestinal Tract Management	Supportive strategies with some evidence	Direct intestinal protective strategies are experimental. Beneficial practices include early enteral nutrition, when possible, appropriate antimicrobial prophylaxis, and managing gastric pH. Maintaining gut barrier function may prevent late complications, but optimal methods need more research.	[129,130,131,132]
Mesenchymal Stem Cell Therapy	Experimental; early trials show safety	MSCs can modulate immune responses and promote tissue repair. Early trials indicate safety in allogeneic use with some positive effects on inflammatory markers. Practical challenges include preparation time, cost, and delivery methods, and limiting current clinical applications. Further research is needed before widespread use.	[133,134]
Extracorporeal Cytokine Removal Systems	Experimental; practical challenges limit use	These systems can remove inflammatory mediators but lack clear evidence of improving patient outcomes. High complexity, cost, and uncertain timing reduce their utility. More research is required to establish patient selection and optimal intervention timing before recommending routine clinical use.	[135]
Integration of Therapeutic Approaches	Essential for optimal outcomes	Coordinated application of interventions across different phases is crucial. Early focus on hemorrhage control and resuscitation per damage control principles shows survival benefits. Timing and sequence of interventions, tailored to injury patterns and severity, are as important as the treatments themselves.	[75,136]
Therapeutic Intensity Adaptation	Based on injury pattern and shock severity	Treatment intensity should adapt to the specific injury and shock level. Penetrating trauma may require aggressive resuscitation, while blunt trauma may need more focus on inflammation modulation. Transitioning between treatment phases relies on clinical and laboratory monitoring to guide adjustments.	[52,53]
Balancing Organ Support Strategies	Requires individualized approach	Fluid management must balance preventing pulmonary edema with ensuring renal perfusion. Anticoagulation for prevention of thrombosis must consider bleeding risks. Standardized protocols need flexibility to cater to individual patient needs, representing a challenge in management.	[119,126,137]
Critical Assessment of Therapeutic Evidence	Importance of evidence quality and applicability	Strongest evidence supports early hemorrhage control and resuscitation interventions. Molecular therapies often lack robust clinical evidence. Recognition of evidence limitations should guide clinical decisions and future research. Practical implementation challenges like cost and complexity should be considered in therapeutic development.	[102,138,139]

## Data Availability

No new data were created or analyzed in this study.
